# Case Report: FAPI PET/CT detected occult contralateral DCIS and supraclavicular metastasis, guiding neoadjuvant therapy in synchronous bilateral breast cancer

**DOI:** 10.3389/fonc.2026.1816302

**Published:** 2026-04-24

**Authors:** Lin Cong, Bin Ji, Yiqi Gu, Yilin Wu, Wan Wang

**Affiliations:** 1 Department of Breast Surgery, China-Japan Union Hospital of Jilin University, Changchun, China; 2 Department of Nuclear Medicine, China-Japan Union Hospital of Jilin University, Changchun, China

**Keywords:** breast cancer, case report, FAPI, neoadjuvant therapy, PET/CT

## Abstract

**Background:**

Accurate staging is critical for breast cancer management. While [^18^F]FDG PET/CT is the standard for metabolic imaging, it has limitations in detecting tumors with low glucose metabolism and small regional metastases. Fibroblast activation protein inhibitor (FAPI) PET/CT targets cancer-associated fibroblasts and offers a novel diagnostic avenue.

**Case Presentation:**

A female patient presented with left-sided inflammatory breast cancer. Conventional [^18^F]FDG PET/CT showed uptake in the left breast but missed regional spread and contralateral disease. Conversely, a supplemental [^18^F]AlF-NOTA-FAPI-04 PET/CT revealed intense uptake in the left breast (SUVmax 19.45) and detected occult metastases in the left axillary (SUVmax 15.12) and supraclavicular lymph nodes (SUVmax 14.83). Crucially, it also identified a high-uptake lesion in the right breast (SUVmax 15.06), which biopsy confirmed as DCIS, upstaging the diagnosis to synchronous bilateral breast cancer. Following neoadjuvant chemotherapy, follow-up FAPI PET/CT demonstrated that uptake in the supraclavicular and axillary nodes dropped to background levels, predicting a complete response (CR). Postoperative pathology confirmed CR in the lymph nodes.

**Conclusion:**

This case highlights the superior sensitivity of FAPI PET/CT over FDG PET/CT in detecting low-metabolic malignancies like DCIS and accurately staging regional nodal involvement (including supraclavicular nodes). FAPI PET/CT played a decisional role in upstaging the patient and monitoring neoadjuvant therapy response.

## 1 Introduction

Breast cancer is a heterogeneous disease where accurate staging dictates prognosis and treatment (1). Synchronous bilateral breast cancer (SBBC) and regional lymph node involvement present significant diagnostic challenges (2). Traditional [^18^F]FDG PET/CT relies on glucose metabolism, which often results in false negatives for elusive subtypes like ductal carcinoma *in situ* (DCIS) or small lymph node metastases due to their low glycolytic activity (3).

Fibroblast activation protein (FAP) is highly expressed in cancer-associated fibroblasts (CAFs) within the tumor microenvironment but low in normal tissues (4). FAPI PET/CT has emerged as a promising tool with high tumor-to-background ratios (5). Herein, we report a clinical scenario where FAPI PET/CT identified an FDG-occult contralateral DCIS and supraclavicular metastasis in a patient initially presenting with unilateral inflammatory breast cancer, fundamentally altering the clinical staging and therapeutic monitoring strategy.

## 2 case description

### 2.1 Patient presentation and clinical findings

**Figure 1:**
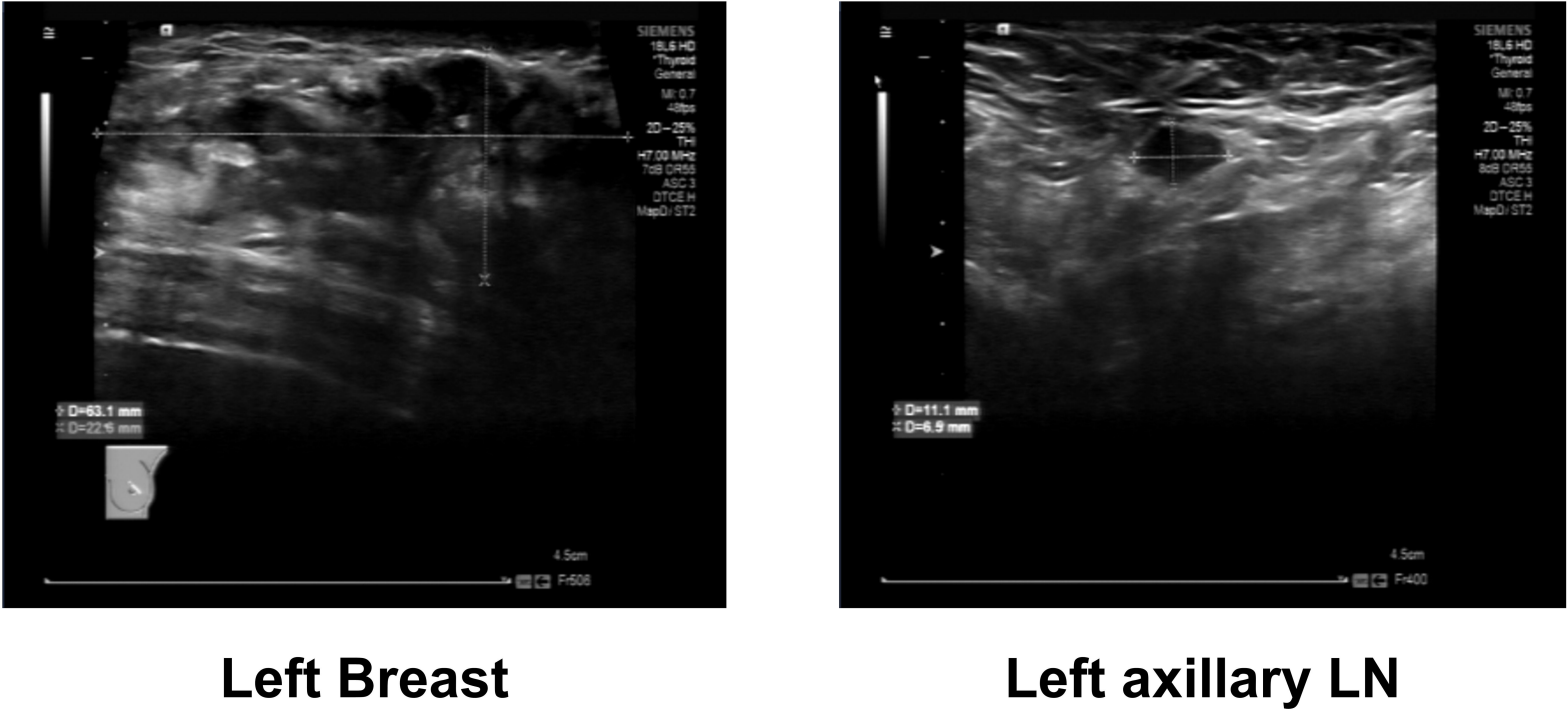
The ultrasound image of the patient's left breast.

**Figure 2:**
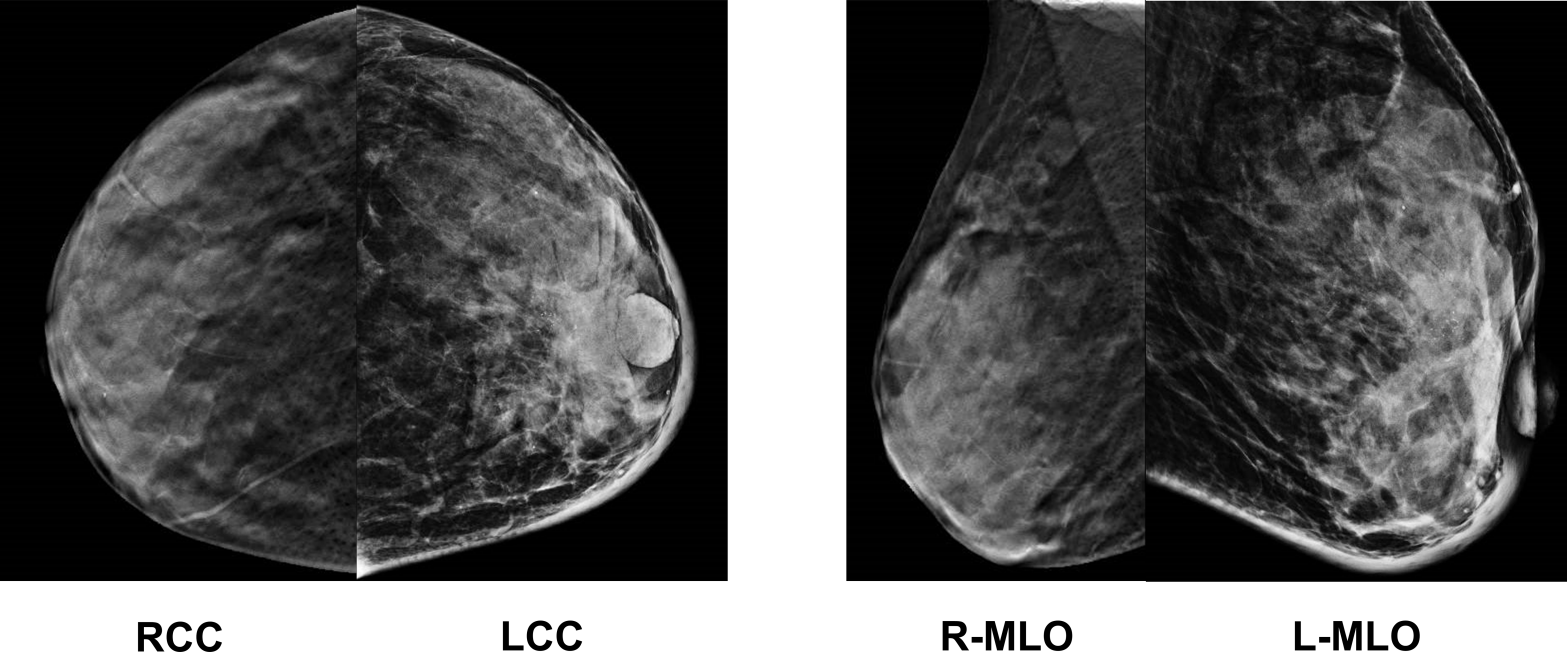
The mammography image of the patient’s breasts.

A 48-year-old female patient presented to our breast surgery department with a primary complaint of progressive redness and swelling of the left breast skin that had persisted for two months. Upon physical examination, the clinical picture was striking: diffuse erythema and edema covered more than one-third of the left breast, accompanied by a distinct “peau d’orange” appearance, a hallmark sign of lymphatic obstruction typical of inflammatory breast cancer (IBC). Although the right breast appeared normal on palpation with no palpable masses or skin changes, the left breast revealed a firm, palpable mass underlying the extensive skin changes. Preliminary ultrasonography (**Figure 1**) identified a hypoechoic irregular mass in the left breast, measuring approximately 6.3*2.3 cm, alongside suspicious cortical thickening in the ipsilateral axillary lymph nodes. Mammography (**Figure 2**) revealed coarse, clustered punctate calcifications under the left nipple, while no significant imaging abnormalities were observed in the right breast. An ultrasound-guided core needle biopsy of the left breast mass was promptly performed, and histopathological analysis confirmed high-grade invasive breast carcinoma. Histopathological evaluation of the ultrasound-guided core needle biopsy from the left breast mass revealed a high-grade (Nottingham Grade III) invasive breast carcinoma. Immunohistochemical (IHC) profiling demonstrated that the tumor is estrogen receptor (ER) positive (80%, 3+), progesterone receptor (PR) positive (60%, 3+), and human epidermal growth factor receptor 2 (HER2) IHC 0, with a Ki-67 proliferation index of 30%. These findings are indicative of a Luminal B (HER2-negative) breast cancer subtype. The initial clinical staging pointed towards a locally advanced, aggressive malignancy requiring immediate systemic evaluation.

### 2.2 Diagnostic assessment

To comprehensively evaluate the extent of the disease and rule out distant metastasis, the patient underwent standard metabolic imaging with [^18^F]FDG PET/CT. The results were partially informative but ultimately incomplete.

At this juncture, a critical diagnostic dilemma emerged. The initial ultrasound examination had explicitly identified suspicious cortical thickening in the ipsilateral axillary lymph nodes, establishing a high morphological suspicion for regional metastasis. The [^18^F]FDG PET/CT results counterintuitively suggested just that, demonstrating a pronounced absence of significant hypermetabolism in the left axillary region. This stark discordance between the alarming sonographic evidence and the deceptively benign metabolic signature on FDG PET/CT rendered current imaging insufficient. To resolve this direct imaging contradiction and firmly establish whether regional metastasis was present, the clinical team decided to utilize FAPI imaging as an advanced problem-solving modality, an examination we were able to offer the patient free of charge thanks to the clinical trials conducted by our nuclear medicine department.

The subsequent [^18^F]AlF-NOTA-FAPI-04 PET/CT scan unveiled a dramatically different and more severe disease landscape. The primary lesion in the left breast exhibited intense tracer accumulation with an SUVmax of 19.45, reflecting a dense, active stromal microenvironment. More critically, the FAPI scan exposed extensive regional nodal involvement that the FDG scan had missed: clearly delineated metastases were visualized in the left axilla (SUVmax 15.12) and, of grave prognostic significance, in the left supraclavicular lymph nodes (SUVmax 14.83).

Perhaps the most pivotal finding, however, was the detection of a completely occult lesion in the right breast. While the FDG scan had shown background-level uptake in the right breast, the FAPI PET/CT highlighted a focal area of intense uptake with an SUVmax of 15.06 (**Figure 2**). This unexpected finding necessitated immediate tissue verification. An ultrasound-guided core needle biopsy of this FAPI-avid right breast lesion was performed. Histopathological examination confirmed the lesion as low-grade DCIS. IHC analysis showed strong and diffuse positivity for ER (90%, 3+) and PR (90%, 3+), HER2 IHC 0, and a low Ki-67 proliferation index of 10%. Consequently, the patient’s diagnosis was fundamentally revised from unilateral IBC to SBBC with extensive regional lymph node metastasis (N3c), a classification that would have been impossible without the superior sensitivity of FAPI imaging.

### 2.2 Therapeutic intervention and follow-up

The revelation of bilateral disease and supraclavicular involvement fundamentally altered the therapeutic trajectory. The initial consideration for immediate surgical intervention was abandoned in favor of a systemic approach to downstage the disease. A rigorous neoadjuvant chemotherapy regimen was initiated. This standardized regimen consisted of 8 cycles and included a treatment plan combining anthracyclines and cyclophosphamide (1/21d * 4) followed by paclitaxel (1/21d * 4), aiming to address both the invasive carcinoma and the high regional tumor burden (6).

**Figure 3:**
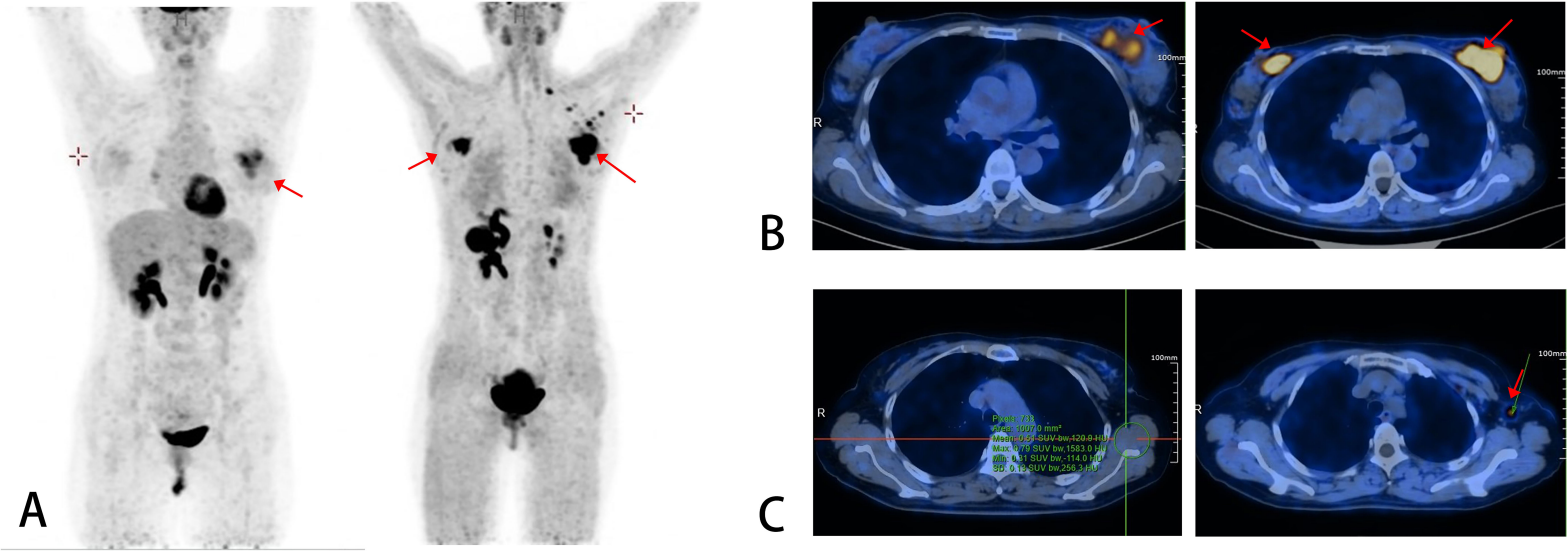
Comparison of two PET/CT images before neoadjuvant therapy. In **(A–C)**, the left images are [^18^F]FDG PET/CT scans, and the right images are [^18^F]AlF-NOTA-FAPI-04 PET/CT scans. The [^18^F]AlF-NOTA-FAPI-04 PET/CT scans demonstrate clear visualization of the primary lesions with high uptake on both sides, as well as the metastatic lesion in the left axillary lymph nodes.

After the completion of eight cycles of neoadjuvant therapy, the patient underwent a comprehensive re-evaluation to assess therapeutic response. The follow-up imaging results (**Figure 3**) provided compelling evidence of treatment efficacy. On the post-treatment FAPI PET/CT, the SUVmax of the left primary lesion plummeted from 19.45 to 2.49, while the occult right DCIS lesion showed a reduction from 15.06 to 1.80. Most notably, the tracer uptake in the previously highly active left axillary and supraclavicular lymph nodes dropped to background levels, suggesting a complete metabolic response in the regional nodes. Interestingly, a comparative follow-up FDG PET/CT showed a paradoxical, slight metabolic increase in the right breast lesion (SUVmax 1.56), a phenomenon interpreted as a chemotherapy-induced “metabolic flare” rather than disease progression, further underscoring the reliability of FAPI in this context.

Following the completion of chemotherapy, the patient underwent a bilateral mastectomy, left axillary lymph node dissection and right sentinel lymph node biopsy. The postoperative pathological examination confirmed the accuracy of the FAPI PET/CT predictions: both the left axillary and supraclavicular lymph nodes showed no viable tumor cells, achieving a pathological complete response (pCR). The primary lesions exhibited significant regression with only residual disease found (Residual Cancer Burden, RCB: 1). The patient’s recovery was uneventful. According to the guidelines, she was subsequently placed on adjuvant intensified treatment with endocrine therapy (letrozole) combined with abemaciclib. By December 2025, she remained in stable condition with no signs of recurrence. **Table 1** presents the complete timeline of the patient’s diagnosis and treatment.

**Table 1:** Timeline of case management.

Timepoint	Clinical event	Key findings/actions
Day 0	Presentation	Patient admitted with symptoms of left inflammatory breast cancer (erythema, edema).
Day 3	Initial Diagnosis	Biopsy of Left Breast: Invasive Carcinoma. Plan: Systemic staging.
Day 5	Imaging 1: [^18^F]FDG PET/CT	Left lesion SUVmax 7.09; Left axilla negative; Right breast negative.
Day 7	Imaging 2: [^18^F]AlF-NOTA-FAPI-04 PET/CT	Right DCIS, Left Axillary & Supraclavicular Metastasis (new findings)
Day 9	Re-evaluation & Biopsy	Biopsy of Right Breast: Confirmed DCIS. Diagnosis revised to Synchronous Bilateral Breast Cancer.
Day 14	Treatment Setup	Initiated Neoadjuvant Chemotherapy cycles.
Month 4	Response Monitoring	Comparative PET/CT scans after NAT.
Month 4	Imaging Results	FAPI PET/CT: Left primary SUVmax dropped to 2.49; Right primary to 1.80; Axilla to 0.97 (background). FDG PET/CT: Right lesion showed paradoxical metabolic increase (metabolic flare) to SUVmax 1.56.
Month 5	Surgery & Pathology	Bilateral Mastectomy. Pathological assessment confirmed therapeutic response (pCR in axilla, partial response in primary lesions).

## 3 Discussion

The clinical course presented here serves as a compelling testament to the limitations of standard metabolic imaging and highlights the distinct diagnostic advantage of FAPI PET/CT in managing complex breast cancer presentations. While [^18^F]FDG PET/CT remains the workhorse of oncologic imaging, its reliance on glucose metabolism can occasionally betray the clinician, particularly when dealing with tumors that exhibit low glycolytic activity or are obscured by inflammatory processes (7). In this specific case of SBBC, the discordance between the aggressive clinical phenotype and the relatively benign findings on FDG PET/CT was striking. How can a locally advanced carcinoma with skin involvement failed to show significant nodal uptake? The answer lies in the biological target: FAPI PET/CT’s ability to visualize the cancer-associated fibroblast-rich stroma provided a clarity that metabolic imaging could not, effectively unveiling the true extent of the disease (5, 8).

Regarding lymph node staging, the superior sensitivity of FAPI PET/CT proved to be the pivotal factor in redirecting patient management (9). Where conventional imaging failed to identify any nodal involvement, FAPI PET/CT unequivocally visualized intense uptake in both the axillary and, more critically, the supraclavicular lymph nodes. This was not merely an academic finding; the accurate detection of supraclavicular metastasis necessitated an immediate upstaging of the disease, shifting the treatment intent from a potentially localized surgical approach to aggressive systemic therapy. Such findings align with emerging evidence suggesting that the dense stromal reaction in metastatic lymph nodes makes them excellent targets for FAPI ligands, even when their metabolic activity is insufficient for FDG detection. Consequently, this case reinforces the argument that for patients with high-risk features, such as inflammatory breast cancer, who present with equivocal or negative FDG findings, FAPI PET/CT should be considered indispensable for ruling out occult regional metastases.

Beyond baseline staging, the utility of FAPI PET/CT extended into the realm of therapeutic monitoring, offering a more reliable biomarker for neoadjuvant response than its metabolic counterpart (10–13). Throughout the neoadjuvant chemotherapy course, the dramatic reduction of SUVmax in the supraclavicular and axillary nodes to background levels correctly predicted the pathological complete response (pCR) that was eventually confirmed surgically. In stark contrast, the FDG PET/CT displayed inconsistent metabolic signals in the right breast, likely attributable to a treatment-induced inflammatory “flare” phenomenon, which could have easily been misinterpreted as disease progression. This divergence underscores a critical clinical lesson: when inflammatory confounding factors are present, the stability of fibroblast-targeted imaging may offer a truer reflection of tumor viability than glucose metabolism.

This case underscores a well-documented limitation of conventional [^18^F]FDG PET/CT in the assessment of certain breast cancer subtypes, particularly those with a pronounced desmoplastic stromal reaction or low glycolytic activity, which can lead to false-negative results in nodal staging (14, 15). We observed that there was an intense [^18^F]FAPI-04 uptake in both the primary tumor and the metastatic lymph nodes, which was overlooked in FDG imaging. This is consistent with many current published studies (13, 16–20), indicating that FAPI has higher sensitivity in detecting the primary lesion and local regional metastasis of breast cancer. The stark contrast between the two imaging modalities in our patient directly illustrates FAPI’s unique ability to target the CAFs that are abundant in the tumor microenvironment, a biological feature independent of glucose metabolism. This aligns with recent studies suggesting that FAPI PET/CT may be especially valuable as a problem-solving tool in scenarios of high clinical or sonographic suspicion coupled with negative or equivocal FDG findings (20–22). Furthermore, the quantitative FAPI uptake values (SUVmax) observed in this case contribute to the emerging evidence supporting the use of FAPI as a potential biomarker for early treatment response monitoring during neoadjuvant therapy, where a lack of significant SUVmax decline might signal chemoresistance and prompt an earlier therapeutic adjustment (11, 23).

**Figure 4:**
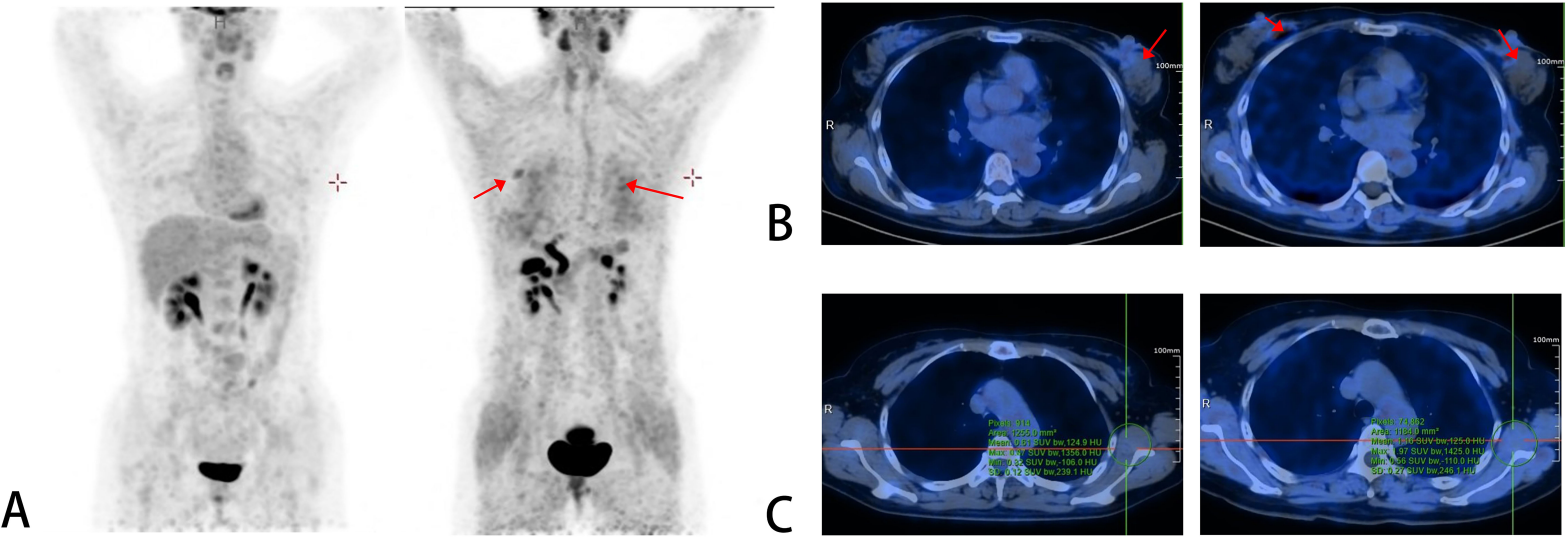
Comparison of two PET/CT images after neoadjuvant therapy. In **(A–C)**, the left images are [^18^F]FDG PET/CT scans, and the right images are [^18^F]AlF-NOTA-FAPI-04 PET/CT scans. Additionally, residual lesions can be observed on the [^18^F]AlF-NOTA-FAPI-04 PET/CT. In both PET/CT scans, the uptake in the left axillary lymph nodes remained at background levels.

Building upon previous studies and observations of this case, we propose incorporating a risk-stratified decision pathway, as illustrated in our conceptual framework (**Figure 4**), into routine clinical practice. This protocol suggests that for patients with FDG-negative results but high clinical suspicion, such as those with inflammatory features or dense breasts limiting mammographic sensitivity, FAPI PET/CT should serve as a second-line screening tool. Furthermore, precise quantification of SUVmax changes during neoadjuvant therapy can act as an early predictor of chemosensitivity. Should the FAPI uptake fail to decline significantly after initial cycles, clinicians might consider an early switch in therapeutic regimens rather than waiting for post-treatment pathological assessment.

Ultimately, this case advocates for the recognition of FAPI PET/CT as a highly decisive problem-solving modality in complex oncological scenarios. By leveraging its ability to accurately visualize the fibroblast-rich tumor microenvironment, clinicians can effectively navigate diagnostic uncertainties and avoid the pitfalls of metabolic inflammatory confounding. This ensures that occult lesions and unexpected disease extensions, such as the distant lymph node involvement and concomitant lesions observed in this patient, are accurately characterized, thereby bridging the gap between imaging limitations and precision oncology.

## 4 Conclusion

In this case of SBBC, FAPI PET/CT acted as a crucial “gatekeeper,” identifying occult supraclavicular metastasis and contralateral DCIS. Its ability to accurately predict pCR in lymph nodes offers a significant advantage for surgical planning and prognostic assessment.

## 5 Patient perspective

“When I was first told I had inflammatory breast cancer, I was terrified. The doctors suggested a new FAPI scan. I was shocked when they told me they found cancer not just in my breast, but also in my collarbone area and the other breast—places the first scan missed. Although hearing the cancer was more widespread was scary, I am grateful it was found. It changed my treatment completely. After chemotherapy, seeing the “glowing spots” in my neck and armpit disappear on the scan gave me huge relief and confidence before surgery.

## Data Availability

The original contributions presented in the study are included in the article/supplementary material. Further inquiries can be directed to the corresponding authors.
